# How to Cope with the Challenges of Environmental Stresses in the Era of Global Climate Change: An Update on ROS Stave off in Plants

**DOI:** 10.3390/ijms23041995

**Published:** 2022-02-11

**Authors:** Archana Singh, Sahil Mehta, Sunita Yadav, Garima Nagar, Rajgourab Ghosh, Amit Roy, Amrita Chakraborty, Indrakant K. Singh

**Affiliations:** 1 Department of Botany, Hansraj College, University of Delhi, New Delhi 110007, India; archanasingh@hrc.du.ac.in; 2 Crop Improvement Group, International Centre for Genetic Engineering and Biotechnology, Aruna Asaf Ali Marg, New Delhi 110067, India; sahil.mehta@icgeb.res.in; 3 School of Agricultural Sciences, K.R. Mangalam University, Sohna Rural, Haryana 122103, India; 4 Department of Botany, Sri Venkateswara College, University of Delhi, Delhi 110021, India; sunitayadav@svc.ac.in; 5 Molecular Biology Research Lab, Department of Zoology, Deshbandhu College, University of Delhi, Kalkaji, New Delhi 110019, India; gnagar@db.du.ac.in; 6 Department of Biotechnology, Amity University, Kolkata 700135, India; rghosh@kol.amity.edu; 7 Excelentní Tým pro Mitigaci (ETM), Faculty of Forestry and Wood Sciences, Czech University of Life Sciences, Kamýcká 129, Suchdol, 165 21 Prague, Czech Republic; Roy@fld.czu.cz; 8 EVA4.0 Unit, Faculty of Forestry and Wood Sciences, Czech University of Life Sciences Kamýcká 129, Suchdol, 165 21 Prague, Czech Republic; 9 DBC i4 Center, Deshbandhu College, University of Delhi, Kalkaji, New Delhi 110019, India

**Keywords:** global climate change, abiotic stress, ROS, signal transduction, antioxidants, ROS scavenging

## Abstract

With the advent of human civilization and anthropogenic activities in the shade of urbanization and global climate change, plants are exposed to a complex set of abiotic stresses. These stresses affect plants’ growth, development, and yield and cause enormous crop losses worldwide. In this alarming scenario of global climate conditions, plants respond to such stresses through a highly balanced and finely tuned interaction between signaling molecules. The abiotic stresses initiate the quick release of reactive oxygen species (ROS) as toxic by-products of altered aerobic metabolism during different stress conditions at the cellular level. ROS includes both free oxygen radicals {superoxide (O_2_^•−^) and hydroxyl (OH^−^)} as well as non-radicals [hydrogen peroxide (H_2_O_2_) and singlet oxygen (^1^O_2_)]. ROS can be generated and scavenged in different cell organelles and cytoplasm depending on the type of stimulus. At high concentrations, ROS cause lipid peroxidation, DNA damage, protein oxidation, and necrosis, but at low to moderate concentrations, they play a crucial role as secondary messengers in intracellular signaling cascades. Because of their concentration-dependent dual role, a huge number of molecules tightly control the level of ROS in cells. The plants have evolved antioxidants and scavenging machinery equipped with different enzymes to maintain the equilibrium between the production and detoxification of ROS generated during stress. In this present article, we have focused on current insights on generation and scavenging of ROS during abiotic stresses. Moreover, the article will act as a knowledge base for new and pivotal studies on ROS generation and scavenging.

## 1. Introduction

**Figure 1:**
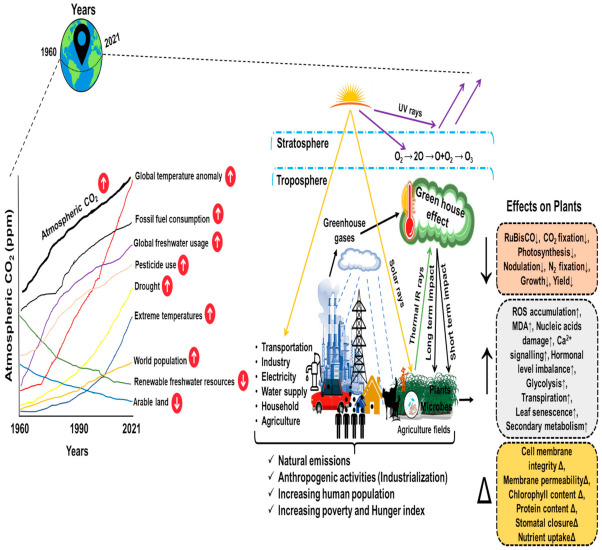
Simplified scheme illustrating the consequences of the current state of climate conditions on environmental threats, plants, and their redox biology arising due to the long-term greenhouse gasses emissions and global temperature anomalies due to anthropogenic activities. Since the age of industrialization, human civilization has impacted the planet Earth, which has become more evident by the effects of anthropogenic activities over the past several decades. These anthropogenic activities in the shade of urbanization, have resulted in the constant increase in greenhouse gas production (primarily CO_2_, CH_4_, N_2_O, H_2_O vapor, and NO). The accumulated CO_2_ in the atmosphere traps the IR radiation emitted from the surface of the Earth following sun rays’ absorption, which heats the planet. As a result, an alarming trend of continual increase in the global surface (and ocean) temperatures has been observed, known as global warming. This global warming, in turn, is driving climate change and decline in ozone levels, which results in increased cases of abiotic stress conditions both in terms of intensity and frequency. At the same time, the overall growth in the exponentially increasing hungry human population is driving an incessant decline in the availability of prime arable farmland and high-quality freshwater for use (graphical trends). Furthermore, the greenhouse gases also exert both short-term and long-term effects on plants’ rate of photosynthesis, stomatal opening, and other cellular activities as well as their microbiome composition (gray box). These processes are further affected by global climate change-driven abiotic stress conditions such as floods, droughts, heat, submergence, etc. Even these climate changes also impact the multidimensions of biotic threats (magnitude, incidence, and population dynamics) on crop plants by changing pest and disease scenarios. For example, the lack of enough duration of temperature below 0 °C favors changes in virulence as well as the appearance of new pests in a region. Moreover, these changes have direct effects on the growth and multiplication, spread, and severity/infestation of many plant pathogens/insect pests. Together, both biotic and abiotic stresses induce the generation of reactive oxygen species (ROS) that eventually reach cytotoxic concentrations and cause oxidative stress in plants (gold circle). To protect themselves, the plant employs a plethora of enzymatic and non-enzymatic antioxidants that alleviate oxidative stress by regulating the antioxidant system. The data presented in the figure has been accessed on 09 February 2022 from Intergovernmental Panel on Climate Change (www.ipcc.ch/), U.S. Environmental Protection Agency (www.epa.gov/), National Centers for Environmental Information (www.ncdc.noaa.gov/), European Environment Agency (www.eea.europa.eu/), and Our World in Data (https://ourworldindata.org/owid-grapher) for the period of 1960–2021. The gold color arrows from the sun depict the sunlight whereas the violet color arrows depict UV rays from the sun, which are reflected by the stratospheric ozone layer. The green-colored arrow depicts the enhancement in levels of greenhouse gases in the troposphere. The broken black line depicts the short-term effects, whereas the black solid line depicts the long-term effects. The delta symbol depicts the altered levels. Different blue brackets are used to differentiate the troposphere and stratosphere of the Earth’s atmosphere from each other. They are depicted here, to represent at both atmospheric strata different climatic changes that occur.

Since the past century, plants have been prone to multiple unfavorable environmental conditions on a time scale of minutes to years [1]. These threats have arisen because of rapid urbanization and anthropogenic activities (including industrialization) that primarily disturb the health of the Earth, plants, animals, and especially humankind. Humans and animals can tackle this extremely serious state because of their ability to move from one state to another. However, in this situation, plants, whether cultivated or growing in wild or forest regions, suffer the most [2]. Therefore, global climate conditions pose a major threat to global food security, influencing agriculture, natural resources consumption, and the exponentially increasing world population (Figure 1). Global agricultural productivity has already been hit by climate change.

In a recent study, Ortiz-Bobea and colleagues found that climate change induced by anthropogenic activities has reduced the world’s agricultural total productivity since 1961 by about 21% [3]. In this respect, the greater impact has been inflicted on the warm and dry regions, including Africa (−34%) rather than cooler regions, such as Europe and Central Asia (−7.1%). Over the coming decades, climate change is expected to have more influence on the world’s food supply. Further, the quantity, quality, and frequency of unpredictable extreme changes such as elevated atmospheric CO_2_, changing temperatures, supercells, cyclones, tides, hails, and rainfall patterns will also impact tremendously [4] (Figure 1).

**Figure 2:**
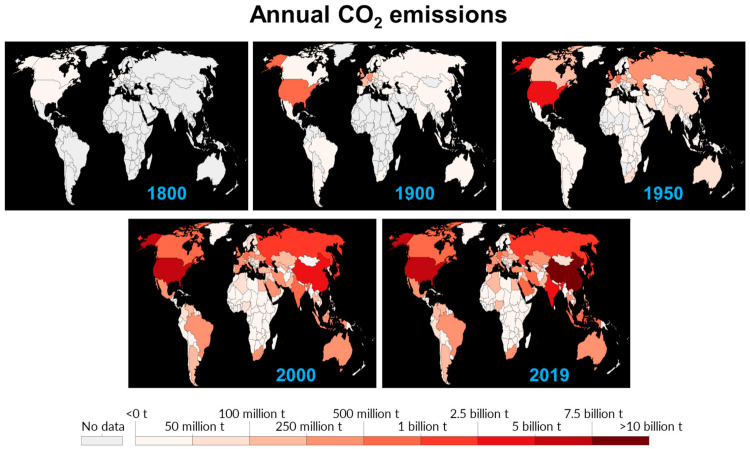
Timescale depiction of annual CO_2_ emissions from the advent of the industrialization era to the year 2019. In this timeline plot, the growth of global emissions before the Industrial Revolution was very low and stayed relatively slow until the mid-20th century. From the year 1950 onwards, the world started emitting a billion tonnes of CO_2_ that have continued to proliferate, with emissions of over 36 billion tonnes per year. In numbers, the world emitted 6 billion tonnes in 1950, 25.23 billion tonnes in 2000, and 36.70 billion tonnes for the year 2019, meaning that emissions have increased sixfold. The most common reason for the significant increases in annual CO_2_ emissions is human activities, i.e., emissions from fossil fuels and land-use change. The data has been accessed on 09 February 2022 from Our World in Data (https://ourworldindata.org/co2-emissions) with Global Carbon Project as a source (https://www.icos-cp.eu/science-and-impact/global-carbon-budget/2021).

Several investigations have reported that a massive increase in the level of the atmospheric CO_2_ concentration has and will alter both plant biomass and biochemistry in most plant species [5], although there are variable responses of individual plant species under elevated CO_2_ conditions. Regarding CO_2_, on the Earth, emissions of CO_2_ and other greenhouse gases are the key regulator of current conditions globally. The data in Figure 2 shows a more drastic impact over the entire time course. Meanwhile, the Earth’s temperature has risen about 1.18 °C/2.12 °F since the end of the 19th century. Consequently, two major ice sheets (Greenland and Antarctic) have declined in mass. Antarctica lost about 148 billion tons, while Greenland lost almost 279 billion tons per year for 26 years (1993 to 2019). Since the emergence of the industrial revolution, an increase in the levels of CO_2_ and other greenhouse gases has directly impacted the acidity of ocean water by 30%. The ocean has absorbed approximately 30% of the total CO_2_ in the past decade, i.e., 7.1 to 10.8 billion metric tons annually). As per the World water data presented in Table 1, the water-related stress and scarcity will be greater in India than in the current most-populous country, i.e., China, by the year 2030. The current climate conditions seem more frightening when we consider the impact of pollution from toxic gases, agricultural waste, industrial runoff, sewage sludge, and many other environmental perturbations on plants’ health.

There are many published data showing the unfavorable impact of global climate change on plant productivity/yield around the world. This in turn also affects the country’s economy in the long run. For example, Abid et al. [6] reported that the variations in the global climate had impacted the livelihoods of the rural families and yield of major food (wheat and rice) and cash (cotton and sugarcane) crops in the Punjab province of Pakistan over the past two decades. Current climate change conditions have impacted Brazilian agricultural production with significant decreases recorded in soybean and corn production. Additionally, the region-wise southward displacement of agricultural production has been also observed in Brazilian biomes [7].

In another report, Rahim and Puay [8] examined the impact of climate change (1983 to 2013) on Malaysia’s economic growth with consideration to precipitation, temperature, and arable land. They observed that the variables are directly related to temperature trends and arable land. Moreover, Dumrul and Kilicaslan [9] found a significant impact of precipitation as well as temperature on the agricultural output in Turkey.

In most parts of China, climate warming has shortened the growth cycle of food crops, which negatively impacts the average production [10]. More recently, Chandio and colleagues explored the long-run and short-run impact of global climate changes on Chinese agricultural output for the period of 1982–2014 [11]. In their study, they concluded that CO_2_ emissions had the most significant effect in both long-run and short-run analyses. Whereas the extreme temperatures and rainfall negatively affected agricultural productivity in the long run. In addition, climate change has also impacted the agriculture productivity in the United States (California), Vietnam, Morocco, India, and sub-Saharan Africa [12,13,14,15,16,17].

At present, there are many forms of such abiotic perturbations that include high and low-temperature stress, salt stress, flooding, dehydration, excess light, heavy metals, ultraviolet radiation, ozone (O_3_), pollution, and many more [18]. A decline in the growth and photosynthetic parameters and reduced germination and vigor is observed during abiotic stress, causing the most deleterious effects on plants and a decrease in crop yield [19,20].

Most laboratories working on abiotic stress focus on the effect of only one or two stress conditions, unlike in nature, where many stresses (“combinatorial stresses”) affect the plants at different points in the life cycle. Across the world, abiotic stress rather than biotic stress has a greater impact on plants, and it can hardly be avoided [21]. Each kind of abiotic stress results in specific signs of damage to the plant due to differences in the signaling pathways and metabolic behavior. However, one of the first plant responses to any form of abiotic stress involves the generation of ROS [22]. Further, ROS activate various secondary messengers, which allow ROS to function as key signaling molecules regulating several pathways in plants. There is a change in the notion that “both plant and animal cells must maintain a balance between ROS generation and its elimination to escape undue oxidation”, and now scientists believe that signaling pathways regulated by a change in concentration of ROS have a positive role to play during stress tolerance [23,24,25].

Photosynthesis and photorespiration are the two main processes that generate most of the ROS. These unavoidable by-products of aerobic metabolism are O_2_^−^, H_2_O_2_, and OH^−^ which are commonly known as ROS. They are produced from molecular oxygen (O_2_) either through incomplete reduction or excitation [26]. ROS have unique biological properties; O_2_^−^ anion, and H_2_O_2_ have preferred biological targets, while hydroxyl radicals react arbitrarily with biological molecules [27]. ROS are produced at low levels in normal physiological conditions; however, in response to various environmental stresses, their concentration dramatically increases, disturbing the homeostasis in the intracellular environment.

ROS has dual functionality in plants. It has been suggested that mild levels of ROS work as a signal to achieve stress tolerance, and at higher concentrations, ROS can lead to cellular damage and programmed cell death (PCD) [28,29,30]. To maintain the equilibrium between the production and removal of ROS, plants have developed scavenging machinery comprised of very efficient enzymatic and non-enzymatic antioxidant defense systems. These defense systems are established in almost all subcellular compartments (chloroplasts, peroxisomes mitochondria, plasma membrane, cell wall, endoplasmic reticulum, and nuclei) to maintain ROS homeostasis in mitigating the process of oxidative stress [31]. A low or moderate concentration of ROS enables them to activate the downstream intracellular signaling cascade by functioning as efficient diffusible secondary messengers [32,33]. The ROS signal can be sensed, transduced, and translated into cellular responses within seconds. Ca^2+^ mobilization and phosphorylation of proteins by Ca^2+^ dependent protein kinases (CDPKs) and mitogen-activated protein kinases (MAPKs), activation of G-proteins, phospholipid signaling, and expression of genes are the various downstream events manifested by ROS. Considering the pivotal role of ROS in abiotic stress, this review highlights the present understanding of the generation and scavenging of ROS in different cellular compartments and their role as signaling molecules.

## 2. Insights into the Basic Mechanism of ROS Generation in Plants

During abiotic stress, various forms of ROSs are generated endogenously within different cellular compartments [34]. Depending on the degree to which the molecular oxygen is reduced, there is a stepwise production of the three main forms of ROS. The primary form of ROS, O_2_^−^, is produced when O_2_ picks up one e^−^. During the respiratory e^−^ transport chain (ETC) in mitochondria and the light-driven photosynthetic ETC, an electrochemical H^+^ gradient for ATP production is established by catalyzing the redox reactions and H^+^ transfer through e^−^ carriers. A highly reduced state can be adopted if the input of energy in the form of light or NADH is high and the availability of intermediate carriers. These carriers include cytochrome c, ubiquinone, and plastoquinone or terminal acceptors (NADP or oxygen) are limited.

NADPH acts as an e^−^ donor, and NADPH oxidase acts as a reaction catalyst. Such conditions lead to the generation of O_2_^−^ at quinones, photosystem I (PSI), and mitochondrial complexes I and III [35]. Further, it could also be released into the apoplast through NADPH oxidase (respiratory burst oxidase homolog, RBOH), present on the plasma membrane. It is known to be involved in local and systemic signaling and stress responses. O_2_^−^ is also produced in the matrix or on the peroxisomal membrane ETC [36].

**Figure 3:**
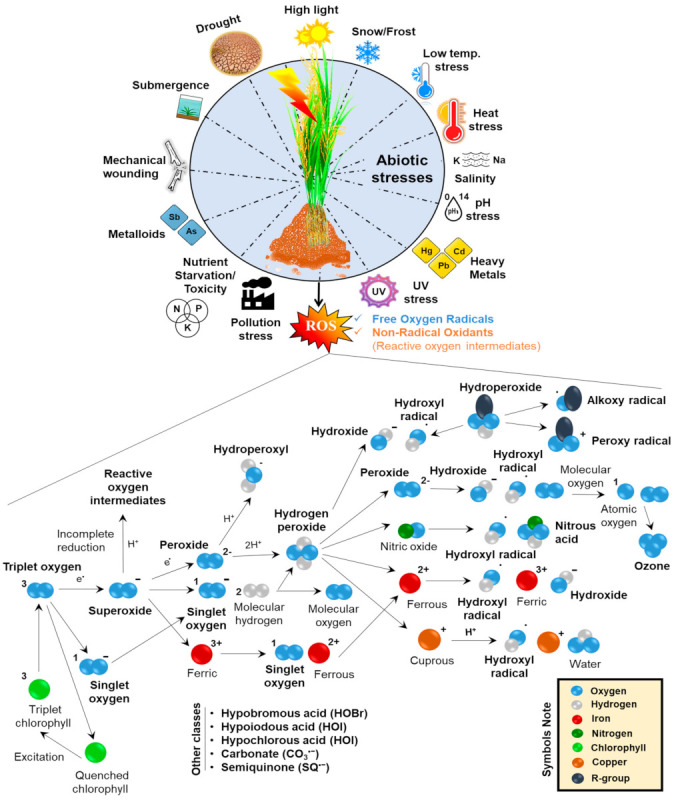
Generation of different forms of ROSs (free oxygen radicals and non-radical oxidants) during environmental stress conditions. In the plant cells, cellular ROS are generated in chloroplasts, mitochondria, peroxisomes, and other compartments. In the chloroplast, the Chl pigments become excited to their triplet state (3Chl*). When this triplet Chl is not quenched efficiently, the triplet oxygen (^3^O_2_) gets excited to singlet oxygen (^1^O_2_). ^1^O_2_ is highly reactive and diffuses outside the chloroplast to reach the cell wall, targets plasma membrane, tonoplast, or even cytosolic signaling cascades. Furthermore, one of the primary forms of ROS, O_2_^−^, is synthesized when ^3^O_2_ accepts an electron (e^−^) from ETC or nicotinamide adenine dinucleotide phosphate NADPH in the presence of NADPH oxidase. In addition, the O_2_^•−^ reacts with H^+^ producing HO_2_^•−^, which is far more reactive, stable, and permeable through biological membranes. Similarly, H_2_O_2_ is formed by dismutation of O_2_^−^/HO_2_^−^ by Superoxide dismutase (SOD) enzymes; first, an intermediate peroxide (O_2_ ^2−^) is synthesized, which is then neutralized by two protons (H^+^) to form H_2_O_2_. Chemically, H_2_O_2_ acts as a weak acid that is highly diffusible and stable. In here, the most important point to be noted is H_2_O_2_ has a longer lifetime than ^1^O_2_ and O_2_^•−^. Another important ROS, OH^−^, is formed by reducing H_2_O_2_ by Fe^+^ and Cu^+^ via Fenton reaction. OH^−^ radicals can also be produced when H_2_O_2_ reacts with superoxide anion (O^•−^_2_), NO, some transition metals, and antioxidants (such as ascorbate). Moreover, RO^•^, ROO^•^, O_3_, HOCl, HOBr, HOI, ROOH, RO*, CO_3_^•−^, and SQ^•−^are incorporated into ROS.

The second form of ROS, H_2_O_2_ is formed by the dismutation of O^•−^_2_ through the enzyme superoxide dismutase (SOD) (O_2_^−^ to H_2_O_2_). O^•−^_2_ is first converted to an intermediate called peroxide (O_2_^2−^) (Figure 3). Two H^+^ then neutralize this intermediate to form H_2_O_2_ (Figure 3). This ROS is a more abundant type because of its low reactivity and high stability [37]. H_2_O_2_ is an essential cellular messenger at low concentrations that relays a signal to provide tolerance against abiotic stress; however, PCD is triggered at higher concentrations [38]. It is produced in peroxisomes mainly through photorespiration as a side product of oxidase reactions such as photorespiratory glycolate oxidase and xanthine oxidase. The inner membrane of the endoplasmic reticulum (ER) might also form H_2_O_2_ due to the catalysis of disulfide bonds in secretory proteins by ER oxidoreductase 1 [39].

The third form of ROS, OH^−^, is the most toxic, as well as the most reactive form. Reduction of H_2_O_2_ by Fe^2+^ and Cu^+^ (redox-active metal ions) through the Fenton reaction results in the production of OH^−^ (Figure 3). H_2_O_2_ can react with O_2_^−^, NO, some transition metals, and also antioxidants such as ascorbate (Asc) to produce the hydroxyl radical [40]. The hydroxyl ion can react with almost all kinds of biological molecules due to its high reactivity, which does not provide it with an efficient detoxification mechanism. However, the hydroxyl radical concentration can be controlled partly by the accumulation of antioxidants such as proline, which are mostly seen to accumulate under stress conditions [41].

^1^O_2_, a form of ROS, is just an excited state of ground-state O_2_, and no gain of e^−^ occurs in its formation. ^1^O_2_ is highly reactive, so much so that, similar to OH^−^, it is thought not to diffuse far from its production site [42]. It is usually generated in the antenna system of the chloroplasts after the 3Chl* reacts with oxygen [43]. It represents the first and foremost excited electronic state of O_2_. However, the ^1^O_2_ is continuously produced during photosynthesis by light-harvesting complexes (LHCs) and reaction centers of PSII. 

During high light stress, photosystem II (PSII) is photo inhibited, significantly increasing the production of ^1^O_2_ in garden pea (Figure 3) [44]. This highly reactive non-radical form is found in the chloroplasts. A sudden increase in light intensity during unfavorable conditions (such as a decline in content of water and CO_2_) results in the production of surplus excitation energy which is not efficiently utilized by photosynthesis. This results in forming a 3Chl*, which upon reacting with ground state oxygen forms ^1^O_2_ [45]. Because ^1^O_2_ degrades pigment and damages D1 polypeptide in the reaction center, it is supposed to be the principal factor responsible for a light-induced decline in PSII activity [46]. The production of radical species causes various primary and secondary deleterious effects. The former includes ROS accumulation, metabolic imbalance, enzyme inhibition, and damage photosystem. On the other hand, the secondary effects include oxidative damage and nutritional imbalance [26,47,48]. In spruce, it was observed that hydrogen peroxide accumulates in the pollen tube apex, liberated from amyloplasts or apoplastic region (working site of SOD and NADPH), whereas major production and localization of O_2_^*−^ is still carried in the mitochondria [49].

Analysis using *Arabidopsis oiwa* mutants revealed ROS accumulation in the central cell as well as in the micropylar region of the female gametophyte. The enhanced concentration of superoxide in mutants might cause phenotypic abnormalities in female gametophytes including egg cell impaired specialization and mitosis arrest in megagametogenesis [50]. Similarly, in *A. thaliana*, MOSAIC DEATH 1 deficiency, a plastid-oriented enoyl- ACP reductase crucial for fatty acid biosynthesis, resulted in alleviated export of malate content from chloroplast to mitochondria and the significant production of PCD and ROS induced via mitochondria. Additionally, mitochondria NAD pool regulation through NAD^+^ transporter II was found to be important for NADH homeostasis in matrix, leading to the production of ROS in *Arabidopsis* [51]. Recent studies revealed that NADH oxidation generates mitochondrial ROS which can initiate a signaling cascade to stimulate energy metabolism and causes PCD [52].

## 3. Effects of ROS and Signaling in Different Cellular Compartments

During abiotic stress, ROS is being released both in light and in dark conditions, and the concentration of ROS and redox state also varies in each cellular compartment depending on the type of stimulus (Figure 3). Abiotic stresses trigger a change in ROS levels and redox signature in specific subcellular spaces and activate further signaling events leading to alteration in gene expression in the nucleus by molecular trafficking via cytosol. Although cytoplasm is not the leading site for ROS production, it acts as a place for ROS signal integration and transmission from one cellular compartment to another during stress. ROSs are generated in cell organelles and apoplasts, reach the cytoplasm first, and then are transmitted to the nucleus. The molecular trafficking also occurs in the reverse direction from the nucleus to the cytosol to other cellular compartments to provide the required stress-responsive proteins for maintaining low/moderate ROS levels and avoiding cellular damage caused by oxidative stress [53]. ROS generated in various organelles affects Chl, ETC, protein, and enzymes. Moreover, affecting a process that controls ROS at the early phase in organelles can restrict further damage. However, ROS channeling into stress-dependent pathways diverts the oxidative injury and promotes the tolerance mechanism [48].

### 3.1. ROS Generation and Signaling in Chloroplasts

During stress, H_2_O_2_ is generated by the reduction of oxygen, which is further transformed to H_2_O_2_ by SODs. Further, H_2_O_2_ is converted to water by ascorbate peroxidases (APXs). Moreover, chloroplast does not rely entirely on stromal and thylakoid APXs to eliminate H_2_O_2_ [54,55,56]. Instead, the APXs work in association with 2-Cys Peroxiredoxins (PRXs) to get rid of ROS (H_2_O_2_) generated during the photosynthetic ETC. ROS buildup is regulated by a complex antioxidant scavenging system involving TRXs, 2-Cys PRXs, SOD, APX, Asc, etc., [57] that function as antioxidants and allow the transmission of oxidative signals [23,24,30].

When leaves are exposed to high light intensity, an increase in the existing capability of photosynthesis is expected to use the captivated energy. Under these conditions, increased production of ROS occurs. Additionally, during stress conditions, stomatal closure is a usual phenomenon that can instigate a reduction in CO_2_ levels, leading to photoproduction of ROS, which enhances ROS concentration in chloroplasts [29]. Oxidative signals generated in chloroplasts are transmitted to the nucleus either directly via stromules or by other means such as direct transfer from nucleus-attached chloroplasts [30].

Abiotic stresses can cause ROS-mediated chloroplast dysfunction and disruption of physiological processes; therefore, chloroplast rescue is needed [55,58]. To cope with oxidative stress, the chloroplasts choose to be equipped with an intricate group of antioxidants that often have overlying and interconnected functions. Carotenoids that are present next to Chl in the light-harvesting complex are very active antioxidants. They can quench the excited Chl and oxygen generated by ROS. Tocopherols are also significant membrane ^1^O_2_ quenchers, which armors the thylakoid membranes to counter lipid peroxidation. ^1^O_2_ signaling has been associated with heat responses [59], but because of its short half-life, it is predicted that it transmits the signal to other intermediates such as β-cyclocitral (BCC) and dihydroactinidiolide (carotenoid oxidative products) [60]. ^1^O_2_ generated in chloroplasts during stress can alter gene expression in the nucleus via a signaling pathway mediated by EXECUTER (EX1) and EX2 [61].

Under stress conditions, intracellular H_2_O_2_ concentration can alter cellular redox regulation causing oxidation of protein thiols. However, H_2_O_2_ was demonstrated to pass through the chloroplast membrane even at reduced concentration and predictably enhanced production of ROS in *Nicotiana benthamina* leaves. These findings depicted the ROS contribution as a messenger molecule in different organelles. More likely, direct H_2_O_2_ movement or transfer from the chloroplast subpopulation is confined to the close proximity of the nucleus and thus overrides control of many gene expression. The exposure of methyl viologen to ROS production locally in chloroplasts of *Arabidopsis* seedlings was recorded. Changes in H_2_O_2_ level and glutamate redox potential with genetic encoding sensors were revealed in mitochondria, chloroplasts, and other cell organelles. These chloroplast-dependent oxidations stimulated ROS production and movement in subcellular compartments that trigger metabolic functions and retrograde signaling to alter genetic controls [62].

During high light stress, isoprenoid precursors such as methyl erythritol cyclodiphosphate (MEcPP) are generated, which serve as a signal for activation in the expression of stress-responsive plastid proteins encoded by nuclear genes [63]. Additionally, chilling stress facilitates solubility and the liberation of O_3_ as well as e^−^ leakage from the reduction of respiratory/photosynthetic ETC that collectively increases ROS production. It also affects enzyme activities, membrane fluidity, decreases tissue water and Chl content in cucumber seedlings. In the tomato plant, accumulation of O_2_^−^ and H_2_O_2_ enhanced the expression of respiratory burst oxidase homologs 1 (RBOH1), reduced Chl florescence, photosynthetic rate, and malondialdehyde (MDA) level when exposed to chilling stress [48,64].

Phosphonucleotide 3′-phosphoadenosine 5′-phosphate (PAP) is a member of a superfamily of nucleotidyltransferases generated in plastids under dehydration and high light intensity. PAP is essential for stress tolerance, and it obstructs the function of exoribonucleases (XRNs), leading to increased expression of stress-responsive genes [48]. PAP is controlled by a phosphatase SAL1, and itself regulates the upregulation of ABA biosynthetic genes and ABA/Ca^2+^ signaling components [65]. SAL1 loses its activity, and the concentration of its substrate increases during ROS generation in the chloroplast, which serves as a hint to begin chloroplast to nucleus retrograde signaling pathways. The H_2_O_2_ scavenger APX2 is also induced by PAP and helps regulate nuclear gene expression that encodes for plastid redox-associated proteins. Therefore, PAP is a good candidate for a mobile signal for a retrograde pathway.

One more chloroplast metabolite, tetrapyrroles [66], are generated by the oxidative breakdown of β-carotene, and also work as a signal during stress-mediated responses. How stress modulates the levels of MEcPP, PAP, tetrapyrroles, and other retrograde signals is still not clear. Altered ROS concentration during stress also leads to an imbalance in photosystem stoichiometry and reprogramming of gene expression, instigating chlorosis and PCD. Antioxidants have an interactive role in free radical production to regulate the interaction between biological molecules and radicals [67].

Another important chloroplastic factor responsible for signal trafficking to the nucleus is serine/threonine-protein kinase 7 (STN7) reported in *Arabidopsis*, which balances excitation between PSII and PSI by adequate phosphorylation of LHC II proteins [68]. Certain proteins are downregulated in the stn7 knockout line such as O_2_^−^ generating respiratory burst oxidase homolog protein D (RBOHD/NADPH oxidase), mitochondrial alternative oxidase (AOX), which induces the cyanide resistant oxidation of ubiquinol and the reduction of molecular oxygen (O_2_) to water (H_2_O), and cytoplasmic thioredoxin H5 (TRX5), a thiol-disulfide oxidoreductase contributed in response to pathogens and oxidative stresses. In contrast, upregulation of monodehydroascorbate reductase 6 (MDHAR6) was observed in *stn7*, which catalyzes the conversion of monodehydroascorbate (MDA) to Asc, oxidizing NADH in the process. These results indicate that ROS generated in chloroplasts can influence the gene expression in the entire cell, and the genes that change their expression need not be related only to photosynthesis.

In contrast, Flu knockout lines show upregulation of AOX and TRX and downregulation of MDHAR6 during ^1^O_2_ signaling. This contrasting feature observed in stn7 (in *Arabidopsis*) and flu is due to the difference in signaling cascade; H_2_O_2_ metabolism occurs in *stn7* [68] while ^1^O_2_ signaling occurs in Flu knockout lines. It has also been reported that a change in ROS concentration in any cell organelle could impact the rate of photosynthesis and lead to the generation of signals that could instigate chloroplast-to-nucleus signaling. During oxidative stress signaling, the serine-threonine protein phosphatase /PP2A shows a negative regulatory role in ROS signaling and mediated ABA regulation in *Nicotiana tobaccum* [31].

Transcription factors are also involved in signaling during ROS stress in the chloroplast, such as NAC domain-containing protein 89 (ANAC089), which binds to the promoter fragment 2 of stromal ascorbate peroxidase (sAPX) that functions as a central element of the chloroplast antioxidant defense system. This finding was dissected using the model *A. thaliana.* Heat stress transcription factors (HSFs), which play a crucial role in a plant’s response to several abiotic stresses by controlling the expression of stress-responsive genes, such as heat shock proteins (HSPs), WHIRLY transcription factor (TFs), constitute one of the vital TF families. They play essential roles in plant metabolism to cope with environmental stresses. The inactive form of ANAC089 is present in the endomembrane compartment, but as soon as ROS is generated during stress, the disulfide bridge of ANAC089 gets converted to sulfhydryl groups, and ANAC089 is released from endomembrane by proteolysis [69]. H_2_O_2_ induces HSF/HSP and chaperones during heat stress in *Arabidopsis* [70].

Heat stress induces HSFA2 in wild-type plants, which is downregulated in the mutant rps1, characterized by disturbed membrane integrity and inhibition of ROS-dependent signaling due to suppression of HSFA2 [71]. Another is mitogen-mediated protein kinase (MAPK)–dependent transcriptional modeling of salinity stress stimulated genes through phosphorylation of transcriptional factors (TFs). For instance, MYB44-like TF and HSFA4A (heat shock TFA4A) are activated by MPK3 and MPK6 phosphorylation. These TFs are activated during heat stress and alleviate the overexpression of HSFA4A that decreases lipid peroxidation and H_2_O_2_ concentration [35].

WHIRLY is a family of multimeric proteins and is a specific sensor of the plastoquinone (PQ) oxidative stress. They exist in association with the thylakoid membranes of chloroplasts. During stress, WHIRLY monomerization can occur, and this monomeric form of WHIRLY works as a signal and can stimulate the expression of defensive genes and a few transcription factors such as WRKY [72,73]. Many putative signals of chloroplast generated by ROS accumulations during abiotic stresses have been deciphered, but the understanding of the entire signaling cascade is still in its primitive state.

Chloroplasts work as antennae, which perceive environmental change and generate signals transmitted to the nucleus to regulate gene expression changes, resulting in stress adaptation/acclimation. ROS signals generated in chloroplasts also regulate epigenetic changes [74] as revealed by a study on MutS HOMOLOG 1 (MSH1), which is a DNA binding protein located in the plastid and mitochondria. The MSH1 protein plays a vital role in the induction of epigenetic stress memories in plants.

Consequently, MSH1-harbouring organelles fall into the category of sensory organelles [75]. Under nutrient deficiency, plants stimulate ROS generation mediated through ethylene signaling pathways. The poor availability of potassium favors ethylene biosynthesis that in turn upregulates ROS production. Other elements such as copper, magnesium, zinc, manganese, etc., are a major part of different enzymes (Mn-SOD, Zn/Cu-SOD, etc.) involved in ROS scavenging and energy metabolism. Such nutrient starvation impairs the capacity of ROS scavenging in different plant species and promotes ROS generation [48]. Excessive ROS levels in chloroplasts cause photo-oxidative stress leading to membrane protein damage and lipid peroxidation that affects the photosystem reaction center and causes cell death [76].

### 3.2. ROS Generation and Signaling in Mitochondria

In mitochondria, high levels of ROS (mainly H_2_O_2_) are generated during environmental stress due to e^−^ escape from complexes I and III of the ETC [77]. During stress, H_2_O_2_, a form of ROS, reacts with Fe^2+^ and Cu^+^ and produces OH^−^ (also known as OH^•^), which is highly toxic and can lead to membrane peroxidation, further causing damage to the entire cell by reacting with nucleic acids, proteins, and lipids [78,79]. DEXH box RNA helicase or mitochondrial pentatricopeptide repeat proteins are involved in ROS synthesis and differential response to ABA. ROS accumulation also occurs due to defective complex I, and non-functional peroxisomal β-hydroxyisobutyryl (HIBYL)-CoA hydrolase (CHY), leading to impaired gene expression during cold stress [80].

The ROS accumulation in the mitochondria during stress conditions is controlled by ascorbate (Asc), a soluble antioxidant synthesized in several steps by many enzymes such as L-galactono-1,4-lactone dehydrogenase (GLDH). ASC is photo protective by nature, and its synthesis is increased during high light conditions. Recently, GLDH was shown to exhibit drought tolerance in wheat [81]. ROS accumulation in mitochondria causes downregulation of auxin-responsive genes, ultimately reducing growth [82]. Moreover, a high concentration of ROS perturbs phosphorylation, causing a reduction in ATP biosynthesis and instigating a reduction in growth. Concomitantly, stress exposure results in the hyper reduction of ETC and e^−^ leakage to O_2_ to ROS family members. For instance, heat stress causes hyperpolarization in winter wheat and accelerates ROS production and respiratory ETC [48,83].

During seed germination, H_2_O_2_ acts as a reactive signaling molecule that significantly passes across biological membranes. The endogenous H_2_O_2_ level increases in seeds during germination via loosening of the cell wall. As H_2_O_2_ production will induce damage to organelles, the resistance mechanism within the cell is initiated [84,85]. Two well-identified H_2_O_2_ responsive genes (*HRG1* and *HRG2*) that play role in H_2_O_2_ production within the cell cause low disruption of the metabolic process [86,87]. In *Arabidopsis*, the signaling network for H_2_O_2_ response gene 1/2 (*HRG1/2*) might immediately act endogenously or exogenously toward H_2_O_2_. The *HRG1/2* knockdown facilitated seedling growth whereas *HRG1/2* overexpression significantly inhibited seed germination. The concentration of ROS in *HRG1* overexpression was remarkably reduced as compared to *HRG1/2* mutants after exposure to H_2_O_2_ treatment in roots [87].

Though we lack direct evidence for the involvement of mitochondrial ROS in signaling, it has been intimated that few transcription regulators such as NAC, ABI4, and WKRY act as a mediator for mitochondrial redox signaling. These transcription factors are known to influence the activation of alternative oxidase (AOX). AOX controls ROS generation in mitochondria by maintaining the active cytochrome C pathway. Similarly, AOX also takes care of chloroplastic fine-tuning under severe drought [88]. The promoter of AOX is negatively controlled by ABI4 (ABA-insensitive phenotype). ABI4 regulates ROS retrograde signaling in the chloroplasts [89,90] indicating a connection between the chloroplasts and mitochondria and nuclear gene expression.

The tolerance against abiotic stress is also regulated by the crosstalk between ABA and ROS signaling networks [91,92]. Although WRKY15 functions as a negative regulator of AOX [93] however, ANAC013 works as a positive regulator, and their overexpression lines are more adapted to oxidative stress than wild type. Thioredoxins (TRXs) serves as a button to operate thiol-disulfide that activates AOX [94,95].

Further, many enzymes of the tricarboxylic acid (TCA) cycle of mitochondria is controlled by the TRX system. TRXs are crucial factors for ROS scavenging. They are present in a broad range of organisms, and their primary function is to reduce the S-S group and modulate proteins by thiol-based redox modifications [96]. The well-known targets of TRXs are peroxiredoxins, glutathione peroxidase, many of the transcription factors, receptors, subunits of complex I in the respiratory chain, and their interactions amongst themselves assist in achieving ROS homeostasis [97]. Further evidence showing the role of TRX during stress tolerance was observed in pea, wherein its overexpression in Tobacco Bright Yellow-2 (TBY-2) cells exhibited increased tolerance towards oxidative stress [98]. TRX helps in H_2_O_2_ scavenging, organic hydroperoxides, and reduces lipids [99,100,101,102]. These findings indicate the role of mitochondrial TRXs as an essential player facilitating mitochondria cytosol cross-talk during stress [53].

An intersecting alteration of genes induced by chloroplast and mitochondrial oxidative stress acting downstream to signaling pathways has been suggested. COX proteins (involved in cytochrome C oxidase assembly) also regulate gene expression in mitochondria under stress conditions. *At*COX17 is upregulated during drought, high salt, UV, and high light conditions, and its overexpression leads to salt tolerance, while silencing of *At*COX17 causes downregulation of stress-responsive genes (*NAC*, *WRKY*, *CAT*, and *AAO*), and a high concentration of ROS is also observed during stress. *At*COX17 proteins are also involved in the alteration of gene expression during chloroplast oxidative stress [103]. Another mitochondrial protein, UCP1, coordinates both respiration and photosynthesis under stress, and its overexpression shows tolerance to biotic and abiotic stresses in tomatoes [104,105] while its downregulation causes a significant reduction in the photosynthetic efficiency [106].

Under salinity/drought stress, the hyper reduction of the mobile ubiquinone (UQ) pool in mitochondria caused by electron paramagnetic resonance (EPR) perturbation leads to ROS generation. In another study, chilling stress induces e^−^ leakage and hyper-reduction that favors the production of free radicals. Concomitantly, high temperature favored hyperpolarization of the inner mitochondrial membrane and disrupted potential gradient that leads to actuate production of ROS through over-reduction of the respiratory e^−^ chain [107]. Similarly, *Medicago truncatula* showed genotype-specific mediation of the interaction between salt tolerance and cellular damage indicators, including H_2_O_2_ and MDA content under salinity stress. Interestingly, 150 mM NaCl exposure to *Ailanthium altissima* had alleviated the activities of the antioxidant enzyme without affecting H_2_O_2_ content. However, e^−^ leakage (EL), lipid peroxidation, and total ROS level were twice when exposed to higher salinity conditions in rice root tissues [102].

### 3.3. ROS Generation and Signaling in Other Cellular Compartments

Abiotic stress is one of the crucial sources of ER stress, which leads to protein misfolding and accumulation of unfolded proteins. As soon as it is realized that there is an accumulation of misfolded proteins, to achieve homeostasis, genes encoding chaperones and other proteins that are involved in protein folding are upregulated. In addition, levels of other proteins necessary for protein degradation and translation suppressors are also amplified to reduce the ER workload [108] ER stress is sensed by sensors: transcription factors associated with ER membrane (bZIP28 during heat stress, bZIP17 during salt stress, and NAC during many forms of abiotic stress) and RNA-splicing factors such as IRE1. IRE1 from *Arabidopsis* can diagnose and splice bZIP60 mRNA, resulting in the formation of a variant of bZIP60, which can enter the nucleus and alter gene expression [109].

The peroxisomes exhibit an increase in the rate of photorespiration, leading to high levels of H_2_O_2_ by glycolate oxidase during abiotic stress in *Arabidopsis* [110] which ultimately can change redox status and influence gene regulation at transcription and translation levels [111].

ROS concentration also increases at the cell wall and in the apoplast during abiotic conditions, leading to cross-linking of cell wall components such as phenolics and glycoproteins, resulting in cell wall stiffening [112]. Apoplastic ROS is generated by NADPH oxidases (RBOHs); this process is also known as ROS burst in which extracellular O_2_ is reduced to O_2_^−^ and cytosolic NADPH works as an e^−^ donor. Class III cell wall peroxidases and polyamine catabolizing amine oxidases are also involved in the process [113]. Plant RBOHs are similar to mammalian RBOHs with the apoplastic oxidase domain and the N-terminal regulatory domain directed to the cytoplasm [102,114].

In another study, ^1^O_2_ generated in different cell organelles, other than chloroplasts, altered enzymatic activities related to lipoxygenase and heme protein. These ROS can also be produced from certain defense molecules or phytoalexins (polycyclic quinone hypericin and psoralene) upon successful pathogenic attack and can accumulate in various plant parts in members of the family Apiaceae [115].

The cell wall can also lose Ca^2+^ during salt stress. Moreover, ROS at the cell wall is predicted to stimulate specific signaling cascades. The sensors of cell wall stress are still not identified. The *Arabidopsis* mutant *sos6* has a cell wall defect due to defective *AtCSLD5* (pectin biosynthesis enzyme), and this mutant is susceptible to oxidative stress and other abiotic stresses [116]. Similarly, cellulose synthase has also been implicated to play an essential role in plant growth under salt stress [117]. Many putative signals of ROS have been deciphered, but understanding the entire cascade is still in its primitive state.

## 4. Cytotoxic ROS Damage Cellular Structure and Function

High levels of ROS can be extremely harmful and damaging because of their elevated reactivity and ability to damage cellular structures under conditions of redox imbalance [28]. All the primary four forms of ROS have a characteristic half-life and an oxidizing potential. Abiotic stress generates a large amount of ROS, causing phytotoxicity and ROS to become highly deleterious and leading to unrestricted oxidative stress that can cause membrane damage, protein degradation, enzyme inactivation, oxidative stress damage to DNA or RNA, and lipid peroxidation [118]. During abiotic stress, overall plant metabolism in terms of physiology, biochemistry, and gene expression is affected. Metabolic ROS is produced due to disordered metabolic activity. It controls metabolic fluxes in the cell and directly alters the redox status of rate-limiting enzymes, thereby changing many metabolic reactions [119]. Additionally, the ROS-derived redox modifications can alter the function of key regulatory proteins and thus, affect transcription and/or translation [120]. In mutants sAPX and tAPX, ROS-responsive genes are repressed under high light, indicating that a higher concentration of ROS may decline its signaling role [55].

A high level of ROS generated during abiotic stresses can cause injury to various cellular compartments. Oxidative stresses cause leaf senescence, leaf abscission, reduced plant growth, and poor seed set [121]. Under high ROS concentration, denaturation of plasma membrane proteins occurs, leading to electrolyte leakage [122]. Photosynthesis is also severely affected by oxidative stress. The photochemical reaction in thylakoid lamellae of the chloroplasts is altered, leading to a change in the e^−^ acceptor side of PSII. As a result, photophosphorylation reactions are also modulated in wheat [123].

Severe ROS production under extreme circumstances leads to cellular injuries in creeping bentgrass [124]. Reproductive processes are also affected during oxidative stress; starting from pollen formation, pollen viability, pollen germination, pollen tube formation, egg formation in the ovule, the normal position of the style and stigma, pollen receptivity by stigma, fertilization, endosperm formation, to embryo growth, all the steps of reproduction are severely affected [125].

Also, excessive light stress-driven ^1^O_2_ generation favors oxidation/cleavage of β-carotene in the photosystem reaction center in the core of grana, liberating numerous carbonyl compounds such as apocarotenoids in wild type and chlorophyll mutant plants [126]. Among these products, dihydroactinidiolides and BCC be volatile but active biological compounds that stimulate operational retrograde signaling. Studying an unbiased advanced genetic screen, ^1^O_2_ production elicited stress responses, changes in nuclear gene expression, growth inhibition in certain mutant plants, and cell death in early seedlings were demonstrated to be regulated via EX1, a nuclear-encoded chloroplast protein.

Additionally, ROS production mediated by waterlogging stress initiated the synthesis of photosynthetic energy inducing e^−^ leakage from the photosynthetic e^−^ transport chain. Such liberated e^−^ can bind with a redundant O_2_ molecule to generate O_2_^*−^ which yields to H_2_O_2_ and OH^−^. Moreover, the membrane integrity can be disturbed by the accumulation of ROS under waterlogging [127].

Increases in ROS levels also alter the stability, biosynthesis, concentration, and homeostasis of various growth factors [128,129] causing cellular damage. The uncontrolled oxidation of the cellular components due to high levels of ROS may result in oxidative stress and eventually cell death.

In C_3_ plants, the production of ROS may occur by glycolate oxidation via PCOC (Photosynthesis carbon-oxidation cyclic) events. Such PCOC demonstrated in C_3_ plants, RuBisCO oxygenase RUBP comprises the main alternative sink of e. regeneration of RUBP, thereby suppressing photoinactivation of photosystems (PSII) in case of low CO_2_ level or decreased redox balance [130]. Such self-propagating and sustained mechanisms of ROS production in each cell are chiefly regulated through the stimulation of RBOHD. 

Under stress conditions, toxicity caused by excessive production of ROS is related to disturbed concentrations of proteins and enzymes. It disrupts vital plant processes as well as negatively mediates denaturation of cellular membranes, redox imbalance, oxidation of lipids, ion leakage, and oxidative DNA damage leading to activation of programmed cell death pathways [131]. In *Hordeum vulgare* L., high accumulation of O_2_ and H_2_O_2_ limits product synthesis in shikimate pathways, disruption of redox balance, favors degradation of lipids, photosynthetic pigments, enzymatic activity, and cell membrane [132,133]. Hence, plants have evolved a complex array of enzymatic and non-enzymatic detoxification systems maintaining the balance between production and scavenging of ROS disturbed by stressful conditions [134].

## 5. Enzymatic Antioxidants for ROS Scavenging

The enzymatic components for detoxification of ROS include a wide range of enzymes such as SOD, catalase (CAT), GR, APX, glutathione peroxidase (GPX), DHAR, MDHAR, and GST [135,136]. The mechanism of action and site of reaction of these antioxidant enzymes has been described below and in Table 2.

SOD regulates the concentration of many ROS by catalyzing the conversion of superoxide into oxygen and H_2_O_2_ and checking the potential toxicity caused due to excessive ROS levels. Most organisms possess varieties of SODs in different cellular compartments. Active participation of SODs results in H_2_O_2_ flux due to H_2_O_2_ concentration gradient, leading to activation of redox-sensitive signaling pathways. Under drought conditions, two *LEA* genes, mainly *LEA1* and *LEA2* were introgressed from *Boea hygrometrica* to *N. tabacum*. The transgenic tobacco lines exhibited higher activity of peroxidase (POX), SOD, and PSII dependent enzymatic activities [47,143,144,145].

CAT is an antioxidant enzyme present in all aerobic organisms. It is known to catalyze H_2_O_2_ into water and oxygen in an energy-efficient manner in the cells exposed to environmental stress. CAT is present in all cellular compartments such as peroxisomes, mitochondria, cytosol, and chloroplast of higher plants. It contains a heme moiety at the active site and converts two H_2_O_2_ molecules to oxygen and water. Multiple molecular forms of CAT isozymes (monofunctional CAT or typical CAT, CAT peroxidase, pseudo-CAT, or Mn CAT) indicate their versatile role within the plant system including cotton [146,147].

GR and tripeptide GSH (γ-Glutamyl-Cysteinyl-Glycine) are the two main components of the ascorbate-glutathione (AsA-GSH) pathway, which is meant for defending cells against oxidative stress. GR is a NAD(P)H-dependent enzymatic antioxidant and efficiently maintains the reduced pool of GSH, a cellular thiol. Both GR and GSH are physiologically connected, and it is their differential concentration in plants that is significant for plant defense operations against oxidative stress [148]. Previously, enhanced cesium stress has been observed to upregulate GSH activity in *Arabidopsis*, stimulating the antioxidant system [147].

APX is a member of the plant type heme peroxidase superfamily. The expression levels of APX are modulated in response to various types of abiotic stresses [149,150]. The mitochondrial and chloroplastic APXs (<30 s) have a shorter half-life (>1 h) as compared to cytosolic and peroxisomal isoforms that make them more sensitive in either low concentrations or the absence of AsA in rice [151]. APX is a crucial ROS-scavenging enzyme, which causes reduction of H_2_O_2_ and protects cells from oxidative stress, and Asc serves as a reductant of H_2_O_2_ in chloroplasts, making it an essential factor for H_2_O_2_ detoxification in photosynthetic organisms. During H_2_O_2_ scavenging, APX converts AsA to monodehydroascorbate (MDAsA), a univalent oxidant of AsA, and AsA works as an e^−^ donor. This MDAsA is reduced to AsA by the action of NAD(P)H-dependent MDAsA reductase. DAsA reductase employs GSH to catalyze DAsA, restoring AsA in the cellular compartment. The oxidized GSH is then restored by GSH reductase. Therefore, it is the contribution of all the AsA–GSH cycle components, which prevents the buildup of toxic levels of H_2_O_2_ in plants [152] another cycle, namely, the water–water cycle of the stroma, also takes part in the detoxification of the antioxidative system (AOS) and the dissipation of the energy of excess photons [146,147].

In the water–water cycle, photoreduction of oxygen to water in PSI by the e^−^ derived from water in PSII occur [153]. APXs can scavenge ROS with Asc, leading to DHA and monodehydroascorbate (MDHA) oxidation through DHA reductase and MDHA reductase, respectively. Such activity of Asc regeneration can yield high Asc for ROS scavenging and ultimately lowers the oxidative damage and manage cellular homeostasis in multiple crop plants including rice [99,154,155]. Therefore, a high concentration of AsA levels is mandatory for having an effective antioxidant system. 

GPX is a member of the non-heme-containing peroxidase family and also works efficiently for H_2_O_2_ scavenging [132]. GPX is necessary and indispensable in the entire defense strategy of antioxidants, primarily O_2_^−^, which is perpetually generated in normal body metabolism, mainly through the mitochondrial energy production pathway (MEPP). The active site of Plant GPXs possesses cysteine residue, which is also found in other members of the non-heme family such as glutathione (GSH) and thiol peroxidase [156]. The GPX-encoding genes are differentially regulated during different types of oxidative stress, such as cold stresses in rice [151]. GPXs also regulate the thiol–disulfide balance and maintain cellular redox homeostasis. GPX expression was detected to be highly upregulated in *Brassica rapa* var. nipposinica during stress tolerance [157]. As mentioned above, GPX works in conjunction with APX to maintain the equilibrium and stress response. GPX shows vigorous activity against free radicals, might be using both TRX and GSH as reducing substrates and will overcome the deleterious effect of H_2_O_2_ [36].

Glutathione S-transferases (GST) are evolutionarily conserved enzymes that are important in detoxifying several xenobiotic compounds. These enzymes catalyze the coupling of GSH to electrophilic substrates, producing compounds that are generally less reactive and more soluble. This enables their elimination from the cell via membrane-based GSH conjugate pumps. The broad substrate specificity of GSTs permits them to protect cells against a range of toxic chemicals [158]. GSTs are well-known ROS scavengers, which detoxify secondary ROS produced when ROS react with cellular components. For instance, GSTs allows the cells to get rid of toxic reactive compounds, 4-hydroxynonenal and cholesterol α-oxide, which are produced during the oxidation of membranes by conjugating them with GSH. Some of the GSTs are also involved in the detoxification of organic hydroperoxides [159]. Upregulation of antioxidant genes such as *GST, POX, SOD,* and *CAT* decreases ROS levels. In addition, SOD activity was increased in cowpea leaves when grown under salt exposure tentatively. This indicated the potential role of SOD in mitigating oxidative stress in cowpea seedlings [160]. Similarly, the alleviating activity of GST is dependent upon the amount and duration of stress exposure [161,162].

Findings have also demonstrated that the induced activities of GST genes by salt, cold, heavy metals, and drought are a dominant mechanism for enhanced tolerance to ROS damage. For instance, in *M. sativa*, the upregulation of the *MsGSTU8* with increased GST activities decreases ROS accumulation by enhancing various antioxidant activities to reduce ROS damage and alleviate ROS regulation [139].

MDHAR and DHAR are the two enzymes, which convert the oxidative form of Asc, monodehydroascorbate (MDHA), back to Ascorbic acid (Asca) in the NAD(P)H-dependent manner. Asc is a potent antioxidant, and it gets oxidized to MDHA and eliminates ROS [163]. In a cell, 10% of the total soluble carbohydrate pool is represented by Asc, which works as an e^−^ donor for the scavenging of ROS in plants. MDHAR and DHAR are crucial components maintaining appropriate Asc levels in the cell [164]. Nearly 100 *GSTs* have been studied in maize, soybean, and *Arabidopsis* with a distinct function. Enhanced MDHAR and DHAR activity was observed in different plant species when exposed to abiotic stress [139,165]. In *Lycopersicum esculentum*, the overexpression of MDAR enhances the tolerance ability for temperature stress [166]. 

Plants with an increased tolerance of drought stress and salinity conditions often have a significantly decreased MDA content and ROS accumulation. In *Nicotiana tabacum,* several polyamine biosynthesis genes (*Nt*SAMDC and *Nt*ADC1) and ROS detoxification genes (*POX1*, *POX2*, *SOD*, and *APX1*) were suppressed in rice and spinach [91,167,168]

## 6. ROS Scavenging by Non-Enzymatic Antioxidants

During the last three decades of the twentieth century, research revealed the various components and modes of action of ROS scavenging systems [169]. H_2_O_2_ may be reduced to highly reactive OH^−^ and O_2_^−^ in the presence of transition metals. The only way to avoid oxidative damage is to control the OH^−^ radical generation since this radical has no known scavengers. Therefore, cells must evolve sophisticated strategies to keep the concentrations of H_2_O_2_, O_2_^−^, and transition metals (Fe and Cu) under strict control. In addition to ROS scavenging enzymes non-enzymatic antioxidants have also been discovered to protect against the toxic nature of ROS. This helps in attaining plant acclimation to various abiotic stresses through ROS homeostasis and signaling mechanisms [24]. The detoxification systems employed for ROSs are compartmentalized because of their unique chemical properties and accumulation in a particular cellular compartment. A specific network of antioxidant systems operated via ROS signaling maintains cellular homeostasis and manages Asc-GSH enzyme activities, followed by tocopherols and carotenoids [67,127,145,170].

**Figure 4:**
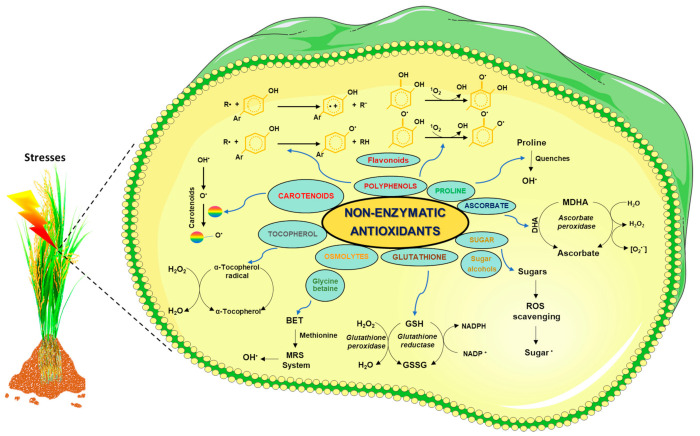
ROS scavenging by various antioxidants to maintain the redox balance. During stress conditions, different cytotoxic ROS family members are scavenged by enzymatic as well as non-enzymatic antioxidants to prevent oxidative damage, otherwise, the lethal ROS family members will cause extensive damage to cellular proteins, nucleic acids, and lipids and thereby affect normal cellular functioning. Herein, the colored ball depicts carotenoids.

To defend themselves from abiotic stresses, many organisms build up compatible solutes (also known as osmolytes). Osmolytes are low-molecular-weight water-soluble compounds such as betaines (glycine betaine), sugars/sugar alcohols (mannitol, sorbitol, and trehalose), polyols, polyamines, Asc, glutathione (GSH), α-tocopherol, alkaloids, flavonoids and carotenoids, and amino acid (proline) [171]. The non-enzymatic antioxidants buildup is preferred under drought or high salt conditions since they offer resistance without disturbing cellular machinery. The oxidized forms of Asc and GSH function as key players among many types of antioxidants available because of their rapid regeneration by particular enzymatic peroxidases and reductases [24] (Figure 4).

Betaine (glycine betaine/GB), an osmolyte, is also synthesized by many organisms to cope with abiotic stress. Chemically, GB is a quaternary amine and carries both positive and negative charges. GB helps in osmotic adjustment and cellular compatibility of the cells that are under stress. GB is synthesized from choline, which gets converted to betaine aldehyde and further to glycine betaine with the assistance of two chloroplastic enzymes, choline monooxygenase, and betaine aldehyde dehydrogenase. The activity of these two enzymes was shown to be upregulated during salt stress. GB alleviates the activity of repair proteins and safeguards the photosynthetic machinery during environmental stresses. In addition, GB facilitates ROS production inhibition, activates stress-related genes, and protects the cellular membrane [172]. In salt-stressed sorghum, reductions were observed in MDA content, polyphenol oxidase (PPO) activity, and H_2_O_2_ when treated with GB. However, Cr stress increased the liberation of ROS and intermediate products (OH^−^ and H_2_O_2_), which enhances the activity of PPO and MDA content in sorghum [173].

Sugars/sugar alcohols (mannitol, sorbitol) that work as antioxidants and provide protection against abiotic stress are of four main types: sucrose, trehalose, raffinose, and fructans [174,175]. Additionally, according to recent reports, inulin, and stevioside also counter against oxidative stress. Sucrose is the key sugar in plant life, and it can be modified to Raffinose family oligosaccharides (RFOs) such as raffinose, stachyose, and verbascose, which are formed in the cytosol [176]. Fructans are also sucrose-derived fructose polymers. The levels of these sugars increase during oxidative stress [177]. This variation in sugar levels leads to a change in the expression of genes involved in stress tolerance, such as SOD, heat shock proteins, and glutathione-S-transferases (GST) [178].

In addition, changes in sugar concentration can also impact Ascorbate (Asc) biosynthesis in harvested broccoli florets [179]. The OH^−^ scavenging ability of galactinol and raffinose has been demonstrated, and their levels are appropriate to protect plant cells from oxidative damage. Among the known sugars, higher fructans concentrations help in overcoming the challenge of salt stress. Conceptually, fructans also scavenge OH^−^, and a derivative of OH^−^ fructans helps in oligosaccharide splitting, which leads to the production of non-radical products. In another study, the concentrations of raffinose and galactose were observed to be lowered in transgenic rice under water-deficient conditions [180]. Further study is needed to gather more information on the chemical identity and stability of sugars and their mechanisms of action during stress tolerance.

Trehalose (Tre) is a non-reducing disaccharide found widely in plants. The disaccharide trehalose, which accumulates dramatically during heat shock and stationary phase in many organisms, enhances thermotolerance and reduces aggregation of denatured proteins [181]. Trehalose can relieve oxidative stress and adjust ion homeostasis under salinity; in addition, trehalose regulates peroxidase and SOD activities to alter plant salt tolerance. Exogenous trehalose has been shown to alleviate ionic imbalance, ROS bursts, and PCD occurrence induced by high salinity in *Arabidopsis* seedlings [182]. In wheat, the exogenous application of trehalose helps to overcome the injuries induced by oxidative stress. Similarly, a mutant of tomato exhibits higher activity of trehalose-6-phosphate synthase under salt stress conditions [180].

Mannitol is a six-carbon non-cyclic sugar liquor and a polyol commonly found in plants and fungi. Mannitol has various capacities: as a carbon stockpiling compound, as a store of reducing power, a compatible osmolyte, and an oxygen radical quencher. Under the circumstances when the sucrose pool is exhausted, mannitol replaces sucrose as translocated sugar in celery [183]. Mannitol protects thiol-regulated enzymes (e.g., Phosphoribulokinase) against hydroxyl radicals that are abundant during the oxidative stress process associated with water stress [184]. Mannitol accumulation can be protective against heat stress-induced oxidative stress. Change in expression of two mannitol genes (Mannitol-1-phosphatase and Mannitol-1 phosphate dehydrogenase) involves ROS signaling, ion homeostasis, ABA signaling, and production of secondary metabolites under different stress conditions in *Arabidopsis thaliana* [185].

Sorbitol is the most commonly found six-carbon sugar alcohol/polyol synthesized during photosynthesis in mature leaves together with sucrose. It translocates carbon and energy from the source to a sink such as sucrose. During high salt and water deficit, the concentration of sorbitol is increased both in the xylem and phloem [186]. Drought-resistant and susceptible varieties of maize exhibited differential antioxidative defense mechanisms [187]. Plants accrue certain sugars (raffinose and sorbitol) to prevent membrane disintegration and enzyme inactivation from diminishing the turgor potential along with detoxification of ROS by reestablishing the cellular redox level [188]. In the case of transgenic potato, enhanced sugar levels resulting from the invertase gene stimulated sugar metabolism enzymes and exhibited resistance to oxidative stress. Moreover, various reports over the past decade have detailed the role of sugars acting as ROS scavengers [179,189].

Asc is involved in redox signaling, regulates the enzymatic activities, provides e^−^ to a large number of reactions that work either enzymatically or non-enzymatically, and modulates gene expression and thus is present in higher amounts than other antioxidants in plants. It also helps in ROS detoxification either directly or through the Foyer–Halliwell–Asada cycle (AsA-GSH cycle), targeting H_2_O_2_ disposal through a cascade of reactions. Asc and GSH [identified in spinach (*Spinacia oleracea* L.)] are part of a complicated antioxidative system [70]. This cycle functions in four different compartments: chloroplasts, mitochondria, peroxisomes, and cytosol, involving four specific enzymes: APX, dehydroascorbate reductase (DHAR), mono dehydroascorbate reductase (MDHAR), and glutathione reductase (GR). It is not the enzymatic component but the antioxidants and NADPH (reducing agent) that seem to be indispensable in the maintenance of the cycle. AsA is found in the cytosol, nucleus, vacuoles, ER, peroxisomes, chloroplasts, and mitochondria. Changes in AsA concentration during abiotic stresses (such as high light, heavy metals, and high O_3_ levels) have also been reported [190].

Asc also protects photosynthetic machinery from high light stress. Asc interacts with plant hormones and regulates plant growth, development, and stress tolerance. The Asc–ABA interaction could involve both ROS-dependent and ROS-independent pathways. Increased Asc concentration will lead to a lower abundance of both ROS as well as ABA. Asc can significantly scavenge ROS and substrate reduction mediated by APXs. However, violaxanthin de-epoxidase (VDE)-like cofactor, directly eliminates ROS [191,192].

GSH is a crucial molecule involved in ROS management, acting as a substrate for some peroxidases and as a reductant of ROS through the Foyer–Halliwell–Asada cycle (AsA-GSH cycle), thus playing critical antioxidant roles in plants [24]. The lighter and non-protein thiol, tripeptide glutathione (γ-glutamyl-cysteinyl-glycine, GSH), plays a key role in intracellular defense against oxidative stress. GSH is present mainly in all organelles and compartments of cell-like apoplasts, cytosol, nuclei, vacuoles, ER, peroxisomes, chloroplasts, and mitochondria. GSH functions as an antioxidant by various mechanisms. It works as a free radical scavenger and scavenges many ROS. GSH also safeguards biomolecules (i.e., proteins, lipids, and DNA) either by glutathionylation or by donating a proton H^+^. Through a thiol–disulfide linkage, the Cys thiol group of GSH (in the reduced state) donates a reducing equivalent to ROS and proteins. Upon removal of hydrogen, a radical form is produced. An oxidized form of GSH known as glutathione disulfide (GSSG) is then formed after the reaction of a GSH radical with another GSH radical.

When most of the GSH of a compartment is utilized while serving as an antioxidant, a change in redox state is gained by Asc-dependent signaling and vice versa. When the stress is raised, GSH concentrations drop, and the redox state becomes more oxidized, which marks the degradation of the system [193]. An increase in the concentration of GSH has been found to provide tolerance against extremes of environmental conditions such as extreme temperature, water stress, salt stress, and heavy metals [99]. In *N. tabacum*, different chloroplast transformants with genes such as *DHAR*, *GR*, and *GSH* show varied antioxidant mechanisms and enhanced abiotic stress tolerance [166].

Polyphenols carry several aromatic groups with phenol and the aliphatic carbon skeleton. Phenolic acids, flavonoids, stilbenes, and lignans are the primary polyphenols [194]. Peroxidases oxidize flavonoids and phenylpropanoids, and H_2_O_2_ is scavenged by the phenolic/AsA/POD system. The phenolic group of polyphenols accepts an e^−^ from ROS and forms stable radicals called phenoxyl radicals, which further inhibit the ROS-induced chain reaction in the cell organelle. Including polyphenols in our diets will strengthen our antioxidant potential since polyphenols themselves get oxidized and spare other components of the cells. Among the notable bioactivities of phenolic compounds, the antioxidant activities have been widely studied, including scavenging free radicals, inhibiting lipid oxidation, and reducing hydroperoxide formation [195]. In *Zea mays,* an enhanced level of vanillic acid was observed under salt stress [196]. Moreover, in tomatoes, the application of vanillic acid enhances ion regulation, osmolyte accumulation, and the antioxidant system [197,198,199].

Flavonoids, an important group of plants polyphenols, have long been known to be synthesized in certain sites and are responsible for the color and aroma of flowers and in fruits to attract pollinators and consequently fruit dispersal to help in seed and spore germination, and the growth and development of seedlings [200]. Additionally, flavonoids play a crucial role as antioxidants during several environmental stresses such as high temperature, water stress, UV radiation, high light, and nutrient insufficiency. Since their discovery, flavonoids have been considered to protect plants against UV radiation by acting as a filter [201]. Currently, flavonoids present in chloroplasts are found to scavenge ^1^O_2_ produced under high light, avoiding cell death. In the presence of light stress, flavonoids concentrate mainly in vacuoles, where they scavenge H_2_O_2_ with the involvement of AsA and peroxidases. Transgenic potato plants with increased concentrations of flavonoids showed improved antioxidant potential [202,203]. In *Salix purpurea*, aglycon naringenin was enhanced under flooding stress while naringenin content was decreased [196].

Tocopherols (α-, β-, γ-, and δ-) are lipophilic antioxidants that scavenge free radicals, lipid peroxyl radicals, and ^1^O_2_. Due to lipid-solubility, antioxidants are highly competent in protecting the biological membranes through the detoxification of radicals of lipids and ROS. Out of the four isomers found in plants, α-tocopherol has the highest antioxidant property [204]. They help in protecting PSII and preventing lipid peroxidation by quenching ^1^O_2_ [205]. An increase in the levels of cellular α-tocopherol (vitamin E) has been found in response to high light and osmotic stress tolerance in tobacco [206]. Similarly, tocopherols and tocotrienols are other key components of the cellular membrane that play a crucial role in the antioxidant system [166,207].

Carotenoids are primarily C_40_ terpenoids, a class of hydrocarbons that take part in several biological processes in plants, such as photosynthesis, photomorphogenesis, photoprotection, and development. Carotenoids also operate as precursors for two plant hormones: lipophilic antioxidants found in both the photosynthesizing and non-photosynthetic tissues of plants. Excess excitation energy is released through the xanthophyll cycle, which helps in protecting the photosynthetic machinery. This results in a reaction with excited Chl molecules, thereby avoiding the production of ^1^O_2_. Increased tolerance towards abiotic stress has been found through a rise in cellular concentrations of carotenoids [208]. In Arabidopsis, three carotene encoding genes (*CAT1, CAT2,* and *CAT3*) were studied during abiotic stress conditions. *CAT1* participates in maintaining cellular homeostasis, whereas *CAT2* is implicated in cold and heavy metal stress. More likely, *CAT3* relays signals during autophagy and PCD in many plants including *Arabidopsis* [209,210,211,212].

Proline, an amino acid, performs a highly beneficial role in plants subjected to several stress conditions. Besides acting as an excellent osmolyte, proline plays three major roles during stress, i.e., as a metal chelator, an antioxidative defense molecule, and a signaling molecule. It also stabilizes the DNA, proteins, enzymes, membranes and helps the cell to conserve energy for future growth and development [168]. Proline can detoxify free radicals and inhibit lipid peroxidation, making it a potential cell death inhibitor and a potent antioxidant. Since the transport of this osmoprotectant occurs between chloroplasts, mitochondria, and cytosol, it has a compartmentalized metabolism. Tolerance to various abiotic stresses such as salinity, drought, heat, and low temperature has enhanced concentrations of this compound [138]. Recent studies showed that proline mutants were drought-sensitive. Proline favors ROS quenching and water uptake, which defends against tissue injury. Instead of scavenging OH^−^ ions, proline attaches redox ion activity and protects from ionic damage. Together with proline, sugars also protect photosystems against peroxidation under water deficit stress [139]. Besides these, a reduction in electrolytic leakage, H_2_O_2,_ and MDA was confined in certain horticulture crops when treated with 0.4% L-cysteine and 15 mM proline under chilling stress [213]. The mechanism is that L-cysteine and proline decrease the permeability of the membrane as well as lowers fatty acid peroxidation at membranal regions. The above finding is well confirmed in barley, citrus, and rice in which exogenous proline exposure reduces MDA content and electrolytic leakage [214,215].

Another metallothionein (MTs) is involved in the detoxification of ROS and regulates redox levels. In *Oryza sativa*, *Os*MT1a expression increased significantly when exposed to drought and salt stress. The alleviated POD, CAT, and APX levels confirmed the improved tolerance of transgenic lines to drought stress [140]. 

## 7. Lower/Moderate Concentration of ROS Works as a Signal during Abiotic Stress

**Figure 5:**
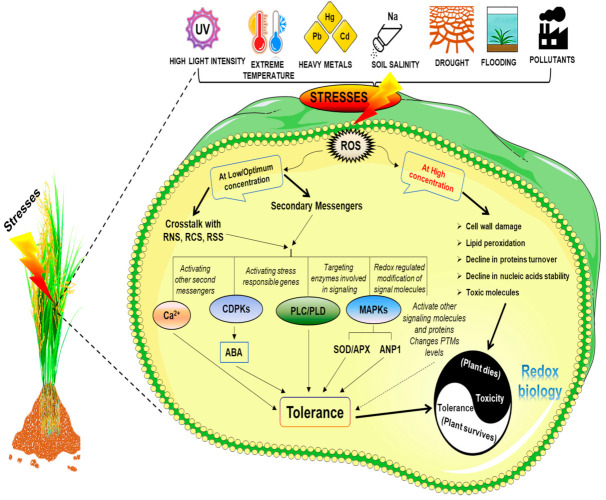
Schematic diagram depicting the signaling pathways activated in response to abiotic stresses in plants. The ROSs crosstalk with vital RNS (reactive nitrogen species), RSS (reactive sulfur species), RCS (reactive carbonyl species), and secondary messengers in plant cells for tackling high oxidative stress by activating various defense mechanisms in plants.

Depending on their cellular amount, ROS has a dual effect under abiotic stress conditions. When kept at relatively low concentrations, they could trigger various stress or acclimatory responses by functioning as a component of the stress signaling pathway [33]. In the signal transduction network, ROS generation is triggered by various stress sensors such as cyclic nucleotide-gated Ca^2+^ channels (CNGCs) [216]. Further, it establishes NADPH oxidases at the plasma membrane, which is either activated by phosphorylation or Ca^2+^ ion fluctuation [217]. The ROS signal can be sensed, transduced, and translated into appropriate cellular responses. Amongst all plant responses, gene regulation is one of the most significant responses towards abiotic stresses such as drought, heat, cold, salt stress, and heavy metals [218]. This leads to unique and universal alterations in genetic machinery at the transcriptional level. Signaling through ROS includes many downstream events such as Ca^2+^ mobilization, activation of G-proteins, phosphorylation of proteins by CDPKs, and MAPK signaling via phospholipids. An alteration in the redox state of regulatory proteins through direct changes in transcription and translation machinery also leads to an acclimation response by ROS signaling (Figure 5). It eventually decreases the amount of metabolic ROS and minimizes the aftereffects of stress on plant metabolism.

A pivotal role of membrane lipids towards signaling in stress conditions is observed, along with its role in modulating the fluidity of membranes in response to various stresses. Signaling molecules are generated from membrane lipids through modifying enzymes such as phospholipases and phosphatases in response to stress. In the presence of various abiotic stresses, a relationship between ROS signaling and lipid signaling is well documented. Phosphoinositide-specific phospholipases are rapidly activated in the presence of various stimuli, leading to the formation of inositol-containing phospholipids called phosphoinositides (PI). This could further help in the formation of molecules involved in signaling pathways. Different environmental stresses can lead to phosphatidic acid (PA) accumulation through enhanced activities of phospholipase C (PLC) or phospholipase D (PLD) enzymes [219]. ABA-induced increased levels of ROS could be observed in the presence of PA along with NO, H_2_O_2_, and NADPH oxidase isoforms [220].

In response to salinity in roots, the glyceraldehyde-3-phosphate dehydrogenase enzyme is reported to be induced in the presence of PA [220]. When exposed to low-temperature conditions, PLC and PLD become activated, leading to the upregulation of many genes in *Arabidopsis* [146]. NADPH oxidases such as RBOHs are also involved in ROS production [221]. RBOHs have EF-hand containing a catalytic domain, and they are activated by Ca^2+^, leading to the oxidative burst [113]. Oxalate oxidase and xanthine dehydrogenase have been found to produce H_2_O_2_ and mediate signaling pathways during stress conditions in the apoplast [81]. In some instances, stress induces hyperaccumulation of both Nitrous oxide and H_2_O_2_ in *Arabidopsis* root tips, leading to enhanced MDA level (an indicator of abnormal root growth and membrane lipid peroxidation). Additionally, 24-epibrassinolide, similar to stress tolerance regulators, elicits enzymatic and non-enzymatic defense responses in *Cucumis sativa* and alleviates antioxidant activity, including GSH, SOD, and CAT that regulate ion homeostasis and confirm stress resistance in *C. sativa* [222]. These findings confirm that precise ROS forms are generated under stress and fine-tuning of the cell redox state is required for appropriate defense responses against abiotic stress (Figure 5).

## 8. Function of RNA-Binding Proteins in ROS Scavenging

With the introduction of microarray technologies and new-generation sequencing methods, many of the widely used model organisms have been studied at genome and transcriptome levels to gain insights into the differential behavior of transcripts incited by increased ROS levels [223,224]. Before translation, mRNAs are processed for maturity, and they bind to various RNA binding domains (RBDs) containing RNA-binding proteins (RBPs). A polyadenylation factor and cleavage and polyadenylation specificity factor 30 (CPSF30) are associated with oxidative stress tolerance in *Arabidopsis* [225]. Oxidative stress also influences another post-transcriptional process i.e., alternative splicing.

During O_3_ stress, two guard cell outward rectifying K^+^ (GORK) mRNA isoforms were reported; one small intron-free mRNA and a larger mRNA along with the introns. Many of the transcripts of mitochondria and chloroplasts are also modified before they are translated into proteins. The above-mentioned RNA editing factors encode pentatricopepetide repeat (PPR) proteins linked to organellar RNA maturation steps such as splicing and cleavage [226]. It has been studied that oxidative stress influences RNA stability. The mRNA of salt overly sensitive (SOS1), a Na^+^/H^+^ antiporter, is not stable under normal growth conditions, but under oxidative stress conditions, its mRNA turns out to be stable in *Arabidopsis* [227], whereas *At*FER1 transcripts become unstable during oxidative stress [228]. The effect of oxidative stress on translation is also under intense scrutiny due to techniques such as density centrifugation and affinity purification. In plants, translation initiation factors, eIF4A/eIFiso4A, work as redox detectors and cause translation inhibition; they form an intermolecular disulfide bond, resulting in the loss of its cap-binding capacity in wheat [229,230]. Oxidative stress also influences many other post-translational modifications (PTMs) such as phosphorylation, SUMOylation, ubiquitinylation, cysteine oxidation, glutathionylation, and methionine oxidation of proteins in *Arabidopsis* [231,232].

Further, the CAT3 was identified as an mRNA-bound protein by three different methods (RNA immunoprecipitation, individual nucleotide resolution crosslinking, and immunoprecipitation (iCLIP)). CAT3 was also designated as an interacting partner of *At*GRP7 (Glycine rich protein) among several other transcripts of genes involved in redox homeostasis (e.g., *CAT2, FER1, WRKY33, GPX1,* and *GOX1*), strengthening the prediction that GRP7 acts as a crucial factor to cope up with oxidative stresses [233]. On the other hand, in *Capsicum annuum*, WRKY6 and WRKY40 served as a positive regulator to heat stress, whereas WRKY27 and WRKY40 negatively regulate thermotolerance by suppressing ROS detoxification. Further investigation to reveal mRNA-bound proteome might improve our knowledge on PTM of the various candidate interactors responding directly or indirectly to ROS [170,234,235].

## 9. Conclusion and Future Perspectives

During the entire course of millions of years of evolution, green plants have evolved without any interference from other eukaryotes, especially humans. However, for the last fifty years, continuous human activities have introduced many contaminants in the environment that altered the ecological balance (Figure 1). These abiotic stresses in Anthropocene have severely damaged the global crop yield characteristics. Therefore, a void in knowledge needs to be filled about what unforeseen challenges plants will face and how they will respond and acclimatize to climate change.

In this aspect, ROS generation under stress conditions seems to be most documented, with various ROS-related signaling components being identified in several crop species. In contrast to the earlier views, it is now increasingly evident that, even under stress conditions, the production of ROS is not necessarily a picture of cellular dysfunction but could represent a signal for adjusting the cellular machinery to the strained conditions. This becomes possible due to the effective balance between the warfare of ROSs detoxification and generation. In this, the molecular cross-interrelationship of ROS with RCS, RNS, and RSS contributes immensely. However, many times, the disbalance between these reactive species results in ROS toxicity and eventually cell death in plant species.

While significant progress has been made to understand the mechanism behind ROS generation and scavenging by using several biochemical as well as omics-associated technologies in recent years. Many gaps and multiple questions remain in understanding how exactly ROS mediates the stress response of plants under current climate adversities. This question becomes more important because of the occurrence of combinatorial stresses in fields.

There is yet a lot to uncover about the ROS signaling initiation, perception, specificity of the generated signal, and how the delicate balance between production and detoxification is controlled in the cell machinery. Despite the recent advances and refinements over time in the subject matter of involvement of ROS in signaling cascades, future studies are required to solve intricate relationships between ROS and abiotic stresses.

For this, the most crucial point is to measure the ROS dynamics accurately in plant tissues. Classic biochemical techniques tend to rely on cell lysis and staining or detection in an ex vivo environment and are prone to errors due to stress inherent in the methodology. On the other hand, advanced methods broadly fall into the categories of biosensors and chemical probes that precisely image and quantify local, subcellular, and global ROS dynamics. In this subject matter, several small-molecule/fluorescent dyes (2’-7’dichlorofluorescin diacetate (DCFH-DA), Amplex Red, dihydrorhodamine 123 (DHR), and dihydroethdium (DHE)) displaying high selectivity, sensitivity, and spatiotemporal resolution are mostly used. Undoubtedly, there is considerable scope for tackling the problems in tracking ROS and redox status markers in stressed plants. One of such problems is the high background fluorescence of plant tissues in vivo that interferes with accurate ROS detection. To tackle this issue, the development of new-generation protein-based ROS sensors targeted to subcellular compartments will enable in vivo monitoring of ROS cues and redox changes in higher plants. This new generation of sensors will be better than the present one in terms of toxicity, data validation, and requirement of endogenous enzymatic factors (e.g., esterases and peroxidases), and calibration. Furthermore, the continued investments will bring improvements in electron paramagnetic resonance as well as nanosensor-based detection of ROS and PTMs in vitro and in vivo.

Moreover, future studies will also see the application of a whole-plant imaging platform for unraveling the ROS dynamics in mature non-cereal plants, which is currently lacking. In addition, instrumentation such as asymmetrical flow field–flow fractionation and nanoflow ultrahigh performance liquid chromatography-tandem mass spectrometry will see substantial improvement in the future for isolating cellular organelles and subcellular fractions from stressed plants, which is not reported yet.

Moreover, future reports will also enrich the knowledge of plant biologists regarding bridge- and tip-points of a reactive interactome for molecules (ROS, RNS, RCS, and RSS). In addition, future publications will comprehend the exact relationship between ROS, melatonin, and phytohormones. In the forthcoming years, more studies will be conducted on studying the underlying tolerance mechanisms in various newly released stress-tolerant varieties of rice (Sahbhagi Dhan, CR Dhan 801, DRR Dhan 42, 43, 44 and 50), wheat (Borlaug 2020, Tijaban-10, BAW 1064, 1158, 1167, and 1169), maize (HQPM-5), and chickpea (IPCL4-14 and BGM4005). Moreover, the work will also be carried out on rice varieties tolerant of submergence (CR Dhan 801, 802 h, Pooja-Sub1, Maudamani-Sub1, Pratikshya-Sub1, and LalatMAS-Sub1), stagnant flooding (Pooja, Sarala, and Gayatri, Kalashree, Varshadhan, and Hansheswari), aerobic conditions (CR Dhan 201, 204, and 207), and high temperature (Lunasanki). For more detailed information, the entire compendium of abiotic stress-tolerant crop varieties is available on the website of ICAR, the National Institute of Abiotic Stress Management (NIASM) [236].

With a long-term goal to enhance the abiotic stress tolerance by utilizing ROS-related pathways, more and more key regulators, mediators, proteins, and interactors need to be identified via multi-omics technology. This will finally help to incorporate the multiple necessary ROS-associated detoxifying genes (as well as QTLs) into the genetic backgrounds of elite cultivars or hybrids to enhance the abiotic stress tolerance on the way to sustainable agriculture. Additionally, the overexpression of antioxidant-encoding genes via transgenic technology has a positive effect on unraveling the dynamic phenomenon of abiotic stress tolerance. However, there is still a major need to evaluate multiple candidate genes for enhancing tolerance under stressful environments. In addition, chemical priming, as well as systems biology approaches, seems to be an attractive alternative. Apart from speed breeding, multiplex-genome editing or base editing via CRISPR/Cas platforms seems another good option that will aid in editing the ROS-detoxifying regulators in less-tolerant cultivars and varieties.

Additionally, it is recommended that additional steps be taken to use weather forecasting more accurately along with underpinning improved irrigation systems in climate-smart farmlands. This will ultimately pave the way for monitoring the super varieties that display optimum growth and yield in farmers’ fields, even under increasing combinatorial stresses.

## Figures and Tables

**Table 1:** Comparative trends of average water availability per human in the 15 most populous countries (including China and India) for the period of 2000–2030. In the present climate change, the availability of good quality freshwater for agriculture use is a necessity. However, the water availability in each country is declining due to the overall population growth pattern and demand for residential and commercial use. This situation will get more worse in many of the most populous countries due to socioenvironmental impacts related to water level changes under future projections of population growth. The data has been accessed on 09 February 2022 from the Water scarcity clock (https://worldwater.io/). The data for the human population per country is based on the data retrieved from Worldometers (https://www.worldometers.info/world-population/population-by-country/).

Country	Population in 2020 (in Millions)	World Population Share (in %)	People Living in Water-Scarce Areas (in %)	Water Stress (in Millions)			Water Scarcity (in Millions)			Absolute Scarcity (in Millions)		
				2010	2020	2030	2010	2020	2030	2010	2020	2030
China	1439.3	18.47	36	140.6	143.8	142.6	175.2	185.4	185.2	310.1	311.4	322.2
India	1380	17.70	33	108.4	153.6	168.9	193.1	201.2	232.2	216.8	262.6	298.1
United States	331.1	4.25	24	25.7	29.8	33.4	25.9	29.4	34.3	45.9	53.2	61.7
Indonesia	273.5	3.51	36	37.4	34.7	36.2	22	51.2	54.2	33.4	44.4	47.8
Pakistan	220.8	2.83	47	12.7	3.7	3.9	7.5	8.5	10.4	67.5	89.9	105.7
Brazil	212.5	2.73	31	13.1	18.5	24	15.3	12.1	13.8	31.8	54.37	57.9
Nigeria	206.1	2.64	19	28.7	40.9	28.3	15.3	19.8	52.6	12.4	18.7	27
Bangladesh	164.6	2.11	33	14.9	14.1	15.4	39.1	28.7	18.7	6	24.8	39.7
Russia	145.9	0.87	19	7.4	7.8	7.8	9.1	11.9	11.7	12.9	15.2	15.4
Mexico	128.9	1.65	52	6.1	7.2	14.1	6.9	9.6	10.2	45.9	55.1	60.6
Japan	126.4	1.62	25	14.6	12.9	12.8	11.5	8.4	6.8	28.4	22.5	22.6
Ethiopia	114.9	1.47	19	12.2	19.8	21.6	8.5	14.2	25.1	5.2	5.9	10
Philippines	109.2	1.41	26	5	12.9	16	9.3	3.3	3.7	18.2	26.2	29.6
Egypt	102.3	1.31	49	0.1	0.1	0.1	0.1	0.2	0.1	31	47.1	53
Vietnam	97.3	1.25	4	1	4.3	3.5	0	0	0	3.7	3.9	4.2

**Table 2:** Enzymatic components of antioxidant defense mechanism and their modes of ROS scavenging in various stresses.

Abiotic Stress	Enzymatic Component	Enzymes Involved	Cellular Compartments	ROS Scavenged	Mode of Action	References
Salinity, Drought, Heat, Cold	SOD	*FeSOD* (*FSD1*) *FeSOD* (*FSD2*) *FeSOD* (*FSD3*) *Cu/ZnSOD* (*CSD1*) *Cu/ZnSOD* (*CSD2*) *Cu/ZnSOD* (*CSD3*) *MnSOD* (*MSD1*)	Chloroplast Chloroplast Chloroplast Chloroplast Cytoplasm Peroxisome Mitochondria	O_2_^.^	O_2_ + O_2_^.^ + 2H^+^ ↓ H_2_O_2_ + O_2_	[137,138,139]
Salinity, Drought, Heat, Cold	CAT	*CAT1*, *CAT2*, *CAT3*	Peroxisome, Chloroplast, Mitochondria, Glyoxysomes, Cytosol	H_2_O_2_	2H_2_O_2_ ↓ 2H_2_O + O_2_	[139,140]

Drought, Salinity, Extreme temperatures, Heavy metals, High light	APX	*APX1, APX2* *APX3* *APX4* *APX5* *APX6* *APX7* *Stomatal APX* *Thylakoid APX*	Cytoplasm Chloroplast Peroxisome Chloroplast Peroxisome Chloroplast Mitochondria Cytoplasm Mitochondria Chloroplast Chloroplast	H_2_O_2_	H_2_O_2_ + 2AsA ↓ 2H_2_O + 2MDHA	[141,142]
Salinity, Osmotic stress, O_3_	MDHAR	*MDHAR1* *MDHAR2* *MDHAR3* *MDHAR4* *MDHAR5*	Chloroplast, Mitochondria Cytoplasm Cytoplasm Mitochondria Cytoplasm Cytoplasm	H_2_O_2_	MDHA + NAD(P)H + H^+^ ↓ AsA + NAD(P)^+^	[131,142]
Salinity, Heavy metals	DHAR	*DHAR1* *DHAR2* *DHAR3* *DHAR4* *DHAR5*	Chloroplast, Mitochondria Cytoplasm Cytoplasm, Chloroplast Cytoplasm, Chloroplast Cytoplasm, Chloroplast	H_2_O_2_	DHA + 2GSH ↓ AsA + 2GSSG	[27,131]
Salinity, Heavy metals, Drought, Low temperature	GR	*GR1* *GR2*	Cytoplasm Chloroplast Mitochondria	H_2_O_2_	GSSG + NAD(P)H ↓ 2GSH + NAD(P)^+^	[131]
Salinity, Cold, Drought	GPX	*GPX1*, *GPX7* *GPX2*, *GPX8* *GPX3* *GPX4* *GPX5* Phospholipid *GPX6*	Chloroplast Chloroplast, Cytoplasm Mitochondria Cytoplasm ER Chloroplast, Mitochondria	H_2_O_2_	H_2_O_2_ + 2GSH ↓ 2H_2_O + GSSG	[131,136]

## Data Availability

Not applicable.

## Abbreviations

ROS: reactive oxygen species, ROSs: reactive oxygen species (in plural context), CDPKs: Ca^2+^-dependent protein kinases, MAPKs: mitogen-activated protein kinases, PSI: Photosystem I, H_2_O_2_: Hydrogen peroxide, ^1^O_2_: singlet oxygen; OH^−^: hydroxyl radical; SOD: superoxide dismutase, Chl: chlorophyll, LHCs: Light Harvesting Complexes, APXs: Ascorbate Peroxidases, PRXs: 2-Cys Peroxiredoxins, EX1: EXECUTER, MEcPP: methyl erythritol cyclodiphosphate, RBOH1: Respiratory Burst Oxidase Homologs 1, MDA: malondialdehyde, PAP: Phosphonucleotide 3′-phosphoadenosine 5′-phosphate, XRNs: exoribonucleases, STN7: Serine/threonine-protein kinase 7, RBOHD: respiratory burst oxidase homolog protein D, AOX: alternative oxidase, TRX5: thioredoxin H5, MDHAR6: monodehydroascorbate reductase 6, ANAC089: NAC domain-containing protein 89, sAPX: stromal ascorbate peroxidase, HSFs: Heat stress transcription factors, HSPs: heat shock proteins, Why: WHIRLY, TFs: Transcriptional factors, HSFA4A: heat shock TFA4A, PQ: plastoquinone, MSH1: MUTS HOMOLOG 1, HIBYL: β-hydroxyisobutyryl, CHY: CoA hydrolase, Asc: ascorbate, Asca: Ascorbic acid, GLDH: galactono-1,4-lactone dehydrogenase, ABI4: ABA-insensitive phenotype, TCA: tricarboxylic acid cycle, TRXs: thioredoxins, UQ: ubiquinone, GB: glycine betaine, PPO: polyphenol oxidase, GST: glutathione-S-transferases, Tre: Trehalose, DHAR: dehydroascorbate reductase, MDHAR: mono dehydroascorbate reductase, GPx: glutathione peroxidase, GR: glutathione reductase, VDE: violaxanthin de-epoxidase, AsA-GSH cycle: Foyer-Halliwell-Asada cycle/1-Ascorbate-glutathione, GSH: Reduced glutathione, GSSG: oxidized glutathione, PCD: Programmed cell death, MTs: metallothionein, CAT: catalase, tripeptide glutathione: γ-Glutamyl-Cysteinyl-Glycine, MDAsA: monodehydroascorbate, GSH: glutathione, MDHA: monodehydroascorbate, MEPP: mitochondrial energy production pathway, CNGCs: cyclic nucleotide-gated Ca^2+^ channels, PLC: phospholipase C, PLD: phospholipase D, RBDs: RNA binding domains, RBPs: RNA-binding proteins, CPSF30: Cleavage, and Polyadenylation Specificity Factor 30, GORK: Guard Cell Outward Rectifying K^+^, PPR: pentatricopepetide repeat, SOS1: Salt Overly Sensitive, PTMs: Post-translational modifications, iCLIP: individual-nucleotide resolution crosslinking, and immunoprecipitation, RBOHs: respiratory burst oxidase homologs, O_3_: ozone, e^−^: electron, ETC: Electron transport chain, EPR: electron paramagnetic resonance, ER: endoplasmic reticulum, PSII: Photosystem II, AOS: antioxidative system, RFOs: Raffinose family oligosaccharides, e^−^: electron, EL: e^−^ leakage, BCC, β-cyclocitral, PCOC: photosynthesis carbon-oxidation cyclic reaction, RuBP: ribulose bisphosphate, RNS: reactive nitrogen species, RCS: reactive carbonyl species, RSS: reactive sulfur species, POX: peroxidase, QTLs: quantitative trait loci, DNA: Deoxyribonucleic acid, DCFH-DA: 2’-7’dichlorofluorescin diacetate, DHR: dihydrorhodamine 123, DHE: dihydroethdium, TBY-2: Tobacco Bright Yellow-2.
